# Prognostic Factors Associated with Successful Salvage Surgery in Recurrent Oral Cancer

**DOI:** 10.3390/diagnostics11061105

**Published:** 2021-06-17

**Authors:** Mateusz Szewczyk, Paweł Golusiński, Jakub Pazdrowski, Wojciech Golusiński

**Affiliations:** 1 The Greater Poland Cancer Center, Department of Head and Neck Surgery, Poznań University of Medical Sciences, Garbary 15, 61-866 Poznań, Poland; jakub.pazdrowski@op.pl (J.P.); wgolus@ump.edu.pl (W.G.); 2 Department of Otolaryngology and Maxillofacial Surgery, University of Zielona Góra, Zyty 26, 65-046 Zielona Góra, Poland; pawel.golusinski@gmail.com

**Keywords:** oral cancer, salvage surgery, recurrent oral cancer, head and neck, successful salvage

## Abstract

Most patients with recurrent oral cancer are not eligible for salvage surgery. Among those who are candidates for surgical salvage, failure rates are high. Given the potential negative impact of salvage surgery on quality of life (QoL)—particularly in unsuccessful interventions—the decision to operate must be weighed carefully. However, the variables associated with successful surgical salvage in oral cancer have not been clearly established. In the present retrospective study, we sought to determine the factors associated with disease recurrence and successful salvage surgery. We evaluated the following parameters in patients (*n* = 261) treated for primary oral cancer at our institution from 2010 to 2017: age; T/N status; perineurial invasion; lymphovascular invasion; extranodal extension; and margin status. In total, 36 patients (33%) were considered eligible for salvage surgery. Four variables were significantly associated with suitability for salvage surgery: early primary T stage, no primary neck disease (N0), no positive margins in the primary resection, and no adjuvant radiotherapy following primary resection. The only variable significantly associated with improved salvage outcomes was negative margin status after the primary tumor resection, underscoring the importance of margin status on treatment outcomes. Additional studies are needed to identify other factors associated with successful salvage surgery in order to better stratify patients according to the likelihood of success, thus potentially avoiding the negative impact on QoL in patients who undergo unsuccessful surgery.

## 1. Introduction

Oral cavity cancer is the most common type of head and neck cancer and the 8th most common cancer worldwide [1]. The prognosis is poor, with less than 60% of patients surviving for more than 5 years [2]. The gold standard treatment is curative-intent surgery. However, up to 47% of surgically-treated patients develop recurrent disease [3,4,5,6]. Salvage surgery is the treatment of choice in these patients, but 5-year overall survival (OS) rates are less than 40% [7,8].

The decision to perform surgery in patients with recurrent disease depends on whether the tumour is considered resectable. However, tumour resectability is often difficult to accurately determine, as reflected by the high failure rates and poor OS in these patients [3]. Moreover, due to the negative impact of salvage surgery on quality of life (QoL), together with the high risk of further relapse, the decision to operate must be weighed carefully based on the likelihood of success, which is largely determined by the characteristics of the preoperative tumour and other patient-related factors [9,10]. However, the variables associated with successful surgical salvage in oral cancer have not been clearly established.

In this context, the aim of the present study was to assess the patient- and tumour-related variables associated with poor outcomes in recurrent oral cancer and to identify the factors that could help to stratify risk in order to determine which patients are most likely to benefit from salvage surgery for recurrent disease.

## 2. Materials and Methods

### 2.1. Patients

We retrospectively evaluated 261 patients, most of whom were males (*n* = 176; 67%) diagnosed and treated for oral squamous cell carcinoma at our institution from 2010 to 2017. The mean (standard deviation (SD)) patient age was 59 (10.9) years (range 26–97). The two main tumour locations were the tongue (*n* = 115, 44%) and floor of mouth (*n* = 93, 36%) (Table 1). Patients with lip cancer or previous oncological head and neck treatment were excluded from the study. Patients with less than 24 months of follow up (except those who died during this period) were also excluded.

All patients underwent primary surgical resection with ≥1 cm tumour-free margins (both lateral and deep). In patients without clinical nodal involvement (N0), elective neck dissection was performed (nodal levels I–III, unilateral or bilateral in midline tumours). In patients with node-positive neck disease, therapeutic neck dissection (I- IV/V) was performed as appropriate.

The following clinical parameters were assessed and registered for all patients: age at diagnosis; disease stage; T status; N status; perineurial invasion (PNI); lymphovascular invasion (LVI); extranodal extension (ENE); and intraoperative and final margin status. We also assessed the type of recurrence (local, regional, and distant) or second primary tumour. Disease-free survival (DFS) and OS rates were calculated.

We evaluated the characteristics of patients with recurrent disease following unsuccessful primary surgery, including the following: recurrent T stage; recurrent N stage, location of recurrence; distant metastases; second primary tumour; surgical margin status (free/positive) in previously treated surgical patients; adjuvant radiotherapy; subsequent recurrence (Table 2).

Salvage surgery was performed in patients who met eligibility criteria. Patients with unresectable disease or comorbidities who ineligible for salvage surgery. Similarly, patients who had undergone radiotherapy within the last 10 years or who presented severe comorbidities were not eligible for radical radiotherapy and chemoradiotherapy. The outcome of the salvage surgery was defined as successful if there were no signs of recurrent disease after a minimum of 12 months following the intervention

### 2.2. Treatment

For the primary tumour, all patients were evaluated by a multidisciplinary team (MDT), which decided whether the patient was a candidate for adjuvant treatment with radiotherapy and chemotherapy. The standard radiotherapy protocol was 60–66 Gy (2.0 Gy/fraction) administered daily from Monday–Friday for 6 to 7 weeks. Eligibility requirements for adjuvant radiotherapy were: stage pT3/4 tumour; close (1–5 mm) surgical margins; positive lymph nodes; and evidence of perineural or vascular invasion. The indication for chemotherapy included positive surgical margins or extranodal extension. The chemotherapy regimen consisted of concurrent, single-agent cisplatin (100 mg/m^2^) administered every 3 weeks.

### 2.3. Recurrent Cases

All cases of recurrent disease were evaluated by the MDT, which determined suitability for radical treatment, which involved either salvage surgery or full dose radiotherapy (only in radiotherapy-naïve patients). Patients considered ineligible for salvage therapy were prescribed palliative radiotherapy or chemotherapy. Patients who developed a second recurrence after radiotherapy were referred to the MDT for further evaluation.

Due to retrospective study design, approval of Research Ethics Board at Poznan University of Medical Sciences was not considered necessary.

### 2.4. Statistical Analysis

Statistical analysis was performed with the Statistica v. 13.1 software (StatSoft, Tulsa, OK, USA). DFS was defined as the time elapsed from surgery until recurrence or last follow up visit. OS was defined as the time period from surgery until death or last follow-up visit. Kaplan–Meier methods were used to estimate survival outcomes and the log-rank test was used to compare survival curves. The following factors were analysed: age, sex, T stage, N stage, presence of perineural invasion, presence of lymphovascular invasion, presence of extranodal extension (ENE), positive surgical margin, and adjuvant therapy. A univariate and multivariate analysis was performed to determine the effects of age, sex, tumour grade, T/N status, perineurial invasion (PNI), lymphovascular invasion (LVI); ENE, and positive surgical margins on recurrence. The Chi-square test was used to evaluate the influence of these variables on survival outcomes. A cut-off of *p* < 0.5 was set to determine statistical significance.

## 3. Results

### 3.1. Patients

Most patients (78%) were diagnosed with early-stage disease, mainly stage T1 (*n* = 80; 30%) or T2 (*n* = 126, 48%). The most common tumor location was the tongue (*n* = 115; %) and 60 of these patients required partial glossectomy, with four cases of subtotal glossectomy and one total glossectomy. In terms of tumour differentiation, most cases (*n* = 161; 61%) were G2. Mean (SD) follow-up was 43 (28.8) months (range 6−145).

In 140 patients (53%), there was no evidence of metastases to the regional lymph nodes (N0). PNI was present in 33 patients (12%), LVI in 21 (8%), and ENE in 44 (16%). Most patients (*n* = 217; 83%) received postoperative radiotherapy and 71 of these patients (27%) also received chemotherapy (Table 1).

### 3.2. Outcomes

**Figure 1:**
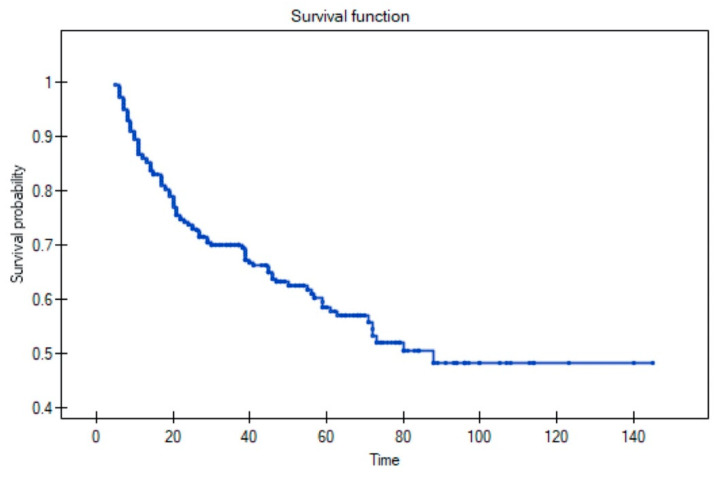
Overall survival rate in full cohort.

During follow-up, 108 patients (41%) developed a recurrence, distributed as follows: local (*n* = 40; 15%), locoregional (*n* = 20; 7%), or regional (*n* = 25; 9%). Of the 20 patients with locoregional recurrence, seven also developed distant disease. Distant metastases were observed in 16 patients (6%), and seven (2.5%) developed a second primary tumour. The 5-year OS rate for the full cohort was 58% (Figure 1).

The mean (SD) time elapsed from primary treatment to recurrence was 20.8 (20.5) months (range 1–90). After primary treatment, 50% of recurrences occurred within the first year and 20% between months 13 to 24.

On the univariate analysis, five variables were associated with a significantly higher risk of recurrence, as follows: nodal disease (*p* < 0.001; hazard ratio (HR) 3.893; 95% confidence interval (CI): 2.310–6.559); PNI (*p* = 0.016; HR 1.193; 95% CI: 1.014–1.244); LVI (*p* = 0.046; HR 1.077; 95% CI: 0.995–1.166); ENE (*p* = 0.003; HR 1.184; 95% CI: 1.048–1.339); and positive surgical margins (*p* < 0.001; HR 3.333; 95% CI: 1.895–5.864). On the multivariate analysis, two variables—positive surgical margins (*p* < 0.001; HR 2.045; 95% CI: 1.380–3.028) and nodal disease (*p* < 0.001; HR 2.326; 95% CI: 1.484–3.646)—remained significant.

Of the 108 patients who developed recurrent disease, 36 (33%) were considered eligible for salvage surgery and the other patients (*n* = 72) were referred to palliative care due to inoperable local and locoregional recurrence (*n* = 50), multiple distant metastases (*n* = 21), and severe comorbidities (*n* = 1). Salvage radiotherapy was not performed in any of the patients.

The mean (SD) time from primary treatment to recurrence in the salvage group was 24.3 (25.8) months (range 1–90). In patients considered ineligible for radical treatment, the mean time from primary treatment to recurrence was 19.1 (17.2) months (range 4 –75).

A univariate analysis was performed to compare patients considered suitable for salvage surgery to those deemed unsuitable, with four variables significantly associated with eligibility for salvage surgery, as follows: early T stage (T1/T2) for the primary tumor (*p* = 0.048); no nodal involvement of the neck (*p* = 0.031); absence of positive margins in the primary resection (*p* = 0.011); and no adjuvant radiotherapy following primary resection (*p* = 0.001). On the multivariate analysis, only T stage (*p* = 0.012; HR 1.433; 95% CI 1.079–1.903) and no adjuvant radiotherapy (*p* = 0.037; HR 0.395; 95% CI 0.164–0.949) remained significant (Table 3).

**Figure 2:**
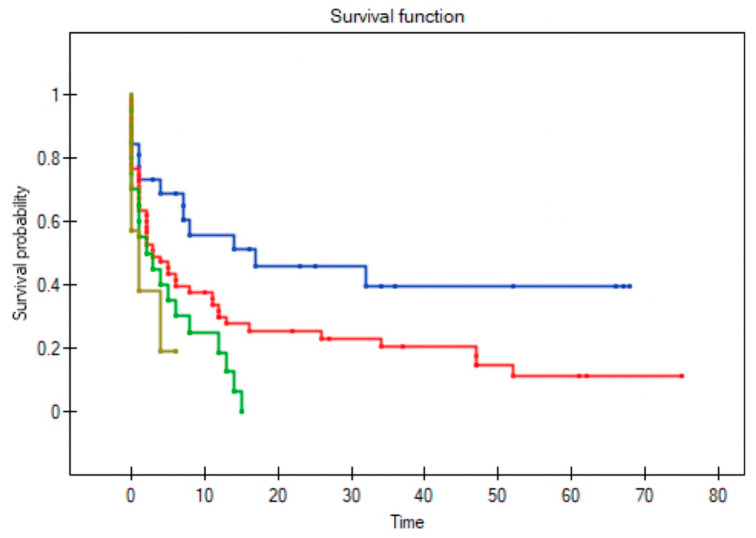
Impact of T stage in the primary resection on survival in patients with recurrent disease. The lines represent stage T1 (blue), T2 (red), T3 (green), and T4 (brown) (log-rank test *p* = 0.004). (Log-rank test *p* = 0.004).

**Figure 3:**
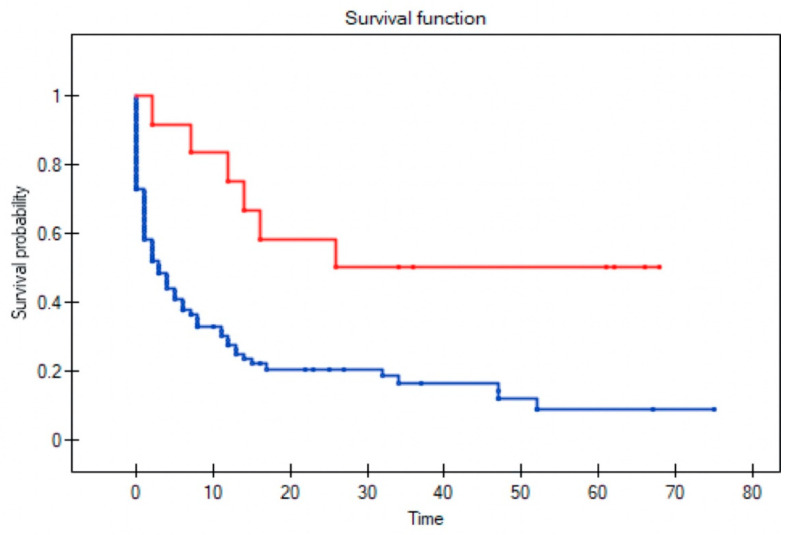
Impact of adjuvant radiotherapy following primary resection on survival in patients with recurrent disease. The lines show survival curves for patients who received adjuvant radiotherapy (red line) versus those who did not (blue line), respectively. (Log-rank test *p* = 0.002).

**Figure 4:**
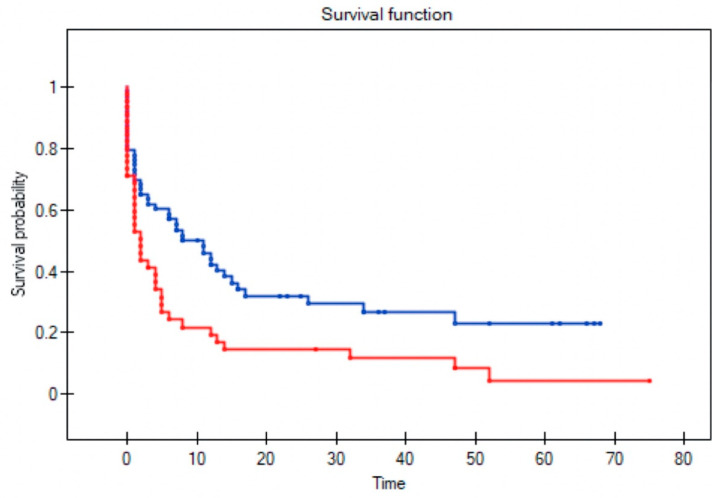
Impact of positive surgical margins in the primary resection on survival in recurrent disease. The lines show survival curves for patients with positive surgical margins (red line) versus those with negative margins. (Log-rank test, *p* = 0.004).

**Figure 5:**
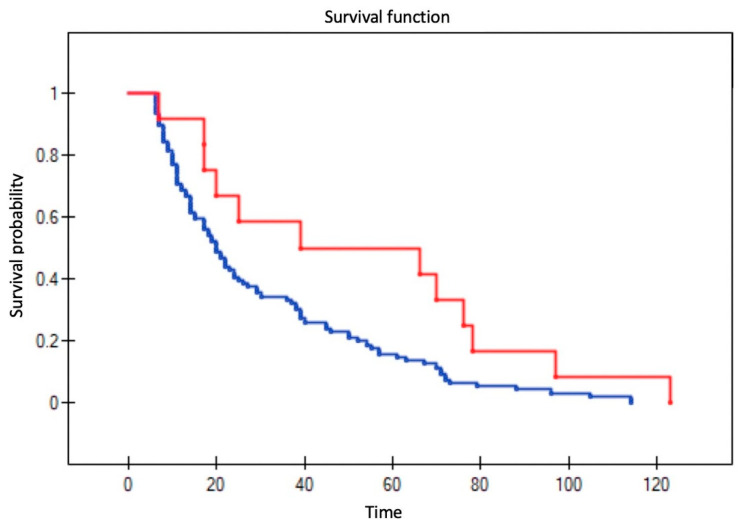
Overall survival in patients with recurrent disease who received adjuvant radiotherapy following primary resection (blue line) versus no adjuvant radiotherapy (red line) (Log-rank test, *p* value = 0.025).

All variables were assessed to determine their influence on survival in patients with recurrent disease; three factors remained significant: T stage (*p* = 0.004); positive surgical margins (*p* = 0.004); and no adjuvant radiotherapy following primary resection (*p* = 0.002) (Figure 2, Figure 3, Figure 4 and Figure 5). Mean survival was significantly longer (80 months) in the patients who underwent salvage surgery than in those who received palliative treatment (40 months; *p* < 0.001; Table 3).

**Figure 6:**
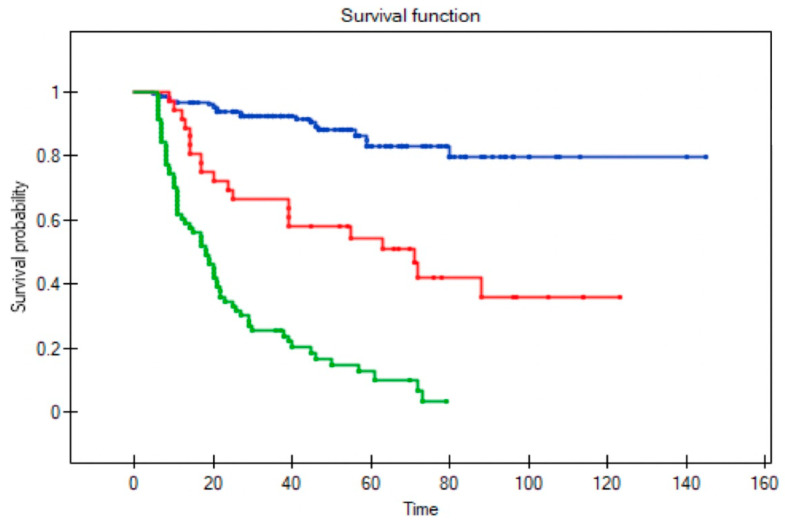
Overall survival in patients without recurrence (blue line), in patients who underwent salvage surgery (red line) and palliative treatment (green line) (Log-rank test *p* < 0.0001).

**Figure 7:**
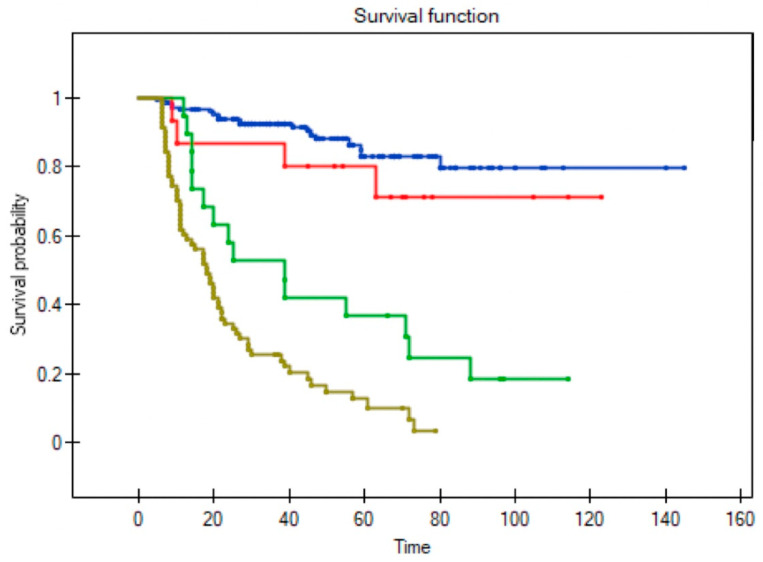
Overall survival in patients with no recurrence (blue line), successful salvage (red line), unsuccessful salvage (green line), and palliative treatment for unsalvageable recurrence (dark yellow line) (Log-rank test; *p* < 0.0001).

Among the 36 patients who underwent salvage surgery, the 5-year OS was 58% and median survival was 52 months (range 9–123). By contrast, in the patients who received palliative treatment, the 5-year OS was only 6% and median survival three months (range 1–27). In the palliative care group, survival was less than six months in 75% of cases (Figure 6 and Figure 7).

Among the 36 patients who underwent surgical salvage, the most common site of recurrence was the tongue (*n* = 14; 36%), requiring subtotal or total glossectomy in 85.7% of cases (12/14). Of the 36 salvage surgeries, 15 (41.7%) were successful (no signs of disease after ≥12 months) and 21 (58.3%) unsuccessful (another recurrence). We evaluated numerous variables (type of recurrence: local, regional, distant, second primary; nodal status, and margin status) to determine which were associated with successful salvage. The only variable significantly associated with improved salvage outcomes was negative margin status in the salvage surgery (*p* = 0.046).

## 4. Discussion

In the present retrospective study, we aimed to determine the factors associated with disease recurrence and successful salvage surgery in patients with oral cancer. On the multivariate analysis, positive surgical margins and nodal disease were associated with a significant increase in recurrence risk. A total of 108 patients developed recurrent disease and, of these, 36 (33%) met criteria for salvage surgery. Four variables were significantly associated with eligibility for salvage surgery: early primary T stage, no primary neck disease (N0), no positive margins in the primary resection, and no adjuvant radiotherapy following primary resection. The only variable significantly associated with improved salvage outcomes was negative margin status after the primary tumour resection.

We found that several factors—N+ neck, PNI, LVI, ENE, and positive surgical margins—were associated with disease recurrence, a finding that is consistent with previous reports [11,12,13,14,15]. In our sample, 108 patients developed disease recurrence a mean of 20.8 months after primary treatment. In most cases (68%), the recurrence occurred within 24 months, underscoring the need for frequent follow up.

This finding is consistent with the study by Brands et al. in 594 oral cancer patients, who found that most locoregional recurrences occurred within the first year and all distant metastases within the first three years [16]. Wang et al. reported similar findings in 312 patients with oral cancer, with a median time to recurrence of 14 months [17]. Interestingly, in some cases the recurrence occurred ≥5 years after primary surgery, suggesting that patients should be offered lifelong follow-up, as recommended by Chou and colleagues [18]. In contrast to other reports, we did not find any correlation between the timing of the recurrence and OS, which was 24.4 months in the salvage group and 19.2 months in the non-salvage groups [5,19]. Mucke et al. analysed large cohort (*n* = 773) of patients with oral cancer, reporting an overall recurrence rate of 23.9%. In that study, patients who relapsed more than 18 months after primary treatment had significantly improved survival versus those with early recurrence. Those authors reported an OS of 31.9% after salvage treatment versus a 5-year OS of 58% in our sample [5]. Weckx et al. also examined time to recurrence and the factors that influence the timing of recurrence in 691 patients with oral cancer. In that study, 60% of patients developed recurrent disease relapsed within first 24 months and patients with early recurrence had significantly worse survival. Those authors also found that timing of recurrence was associated with several variables, including margin status, tumour grade, and lymph node ratio [19].

Given the negative impact of unsuccessful surgical salvage on patient QoL, risk stratification is crucial. In our sample, slightly more than 40% of the salvage surgeries (*n* = 15) were successful versus nearly 60% which were considered unsuccessful. This finding, which is consistent with previous reports, underscores the need to better determine the factors associated with successful salvage therapy. In our study, the only variable significantly associated with salvage outcomes was negative surgical margins in the primary tumour. Borsetto et al. analysed 83 oral cancer patients who underwent surgical salvage in an effort to identify independent predictors of OS, finding that the size of the primary tumour and the primary margin status were the only two significant predictors. Unlike in our study, other independent factors were history of alcohol consumption (not examined in our study) and extent of recurrent disease (non-significant in our study). Those authors also emphasized the technical challenges of salvage surgery and the high treatment-related morbidity rates, which include speech and swallowing difficulties [13].

In borderline cases, we believe that it is essential to evaluate factors to assist with decision-making about possible additional treatments. In this regard, Wecks et al. found an association between negative nodal status (pN) and OS, but only in the univariate analysis [19]. Similarly, Matsuura et al. found shorter survival times (OS and DFS) in patients with nodal involvement in the primary resection [20]. Consistent with previous reports [13,19,21], we found that positive resection margins had a negative impact on OS in patients with recurrent disease.

Salvage surgery following radiotherapy in the primary or adjuvant setting is often challenging due to the presence of fibrosis and scarring. Unsurprisingly, the prognosis in patients who underwent postoperative adjuvant radiotherapy and subsequently developed a recurrence was worse than in those who did not receive radiotherapy, a finding that is in line with previous reports [19,22]. Nevertheless, primary adjuvant radiotherapy has been shown to positively impact survival following salvage therapy [8].

In our patient cohort, of the 36 patients who underwent surgical salvage, only 15 were successful (no signs of residual disease with a minimum of 12 months follow up), with a mean survival of 34 months. By contrast, in the 21 patients whose salvage surgery was considered unsuccessful (another recurrence), the mean survival was only 24 months. In these 36 patients, we evaluated the influence of several variables (T stage, nodal status, and margin status) on outcomes. The only variable associated with survival outcomes was the absence of positive margins in the salvage resection piece. In the study by Tam and colleagues, positive salvage margins were associated with a five-fold increase in mortality risk [8]. Matsura et al. also found that positive surgical margins and the presence of lymph node metastasis (non-significant in our study) had the greatest negative impact on successful salvage [20]. In the study by Kernohan et al. involving 77 oral cancer patients who underwent salvage surgery, the extent of local recurrence within first six months from primary treatment and the extent of nodal recurrence at 6 months following primary treatment were the two factors that that the greatest impact on survival [22].

Our study clearly demonstrates the differences among the various subgroups in terms of survival outcomes. Importantly, the successful salvage group had a similar 5-year OS to that observed in the patients who did not develop a recurrence (80% vs. 82%, respectively). Not surprisingly, the OS rate in the unsuccessful salvage group was substantially lower (38%) and the lowest 5-year OS (<15%) was observed in patients with recurrent disease who received palliative treatment alone. Matsura et al. found that unsuccessful salvaged patients and those who received palliative care had similar survival rates [20].

The main limitation of this study is the retrospective study design. What is more, out of 36 salvaged patients only 41% were successful which could be qualified as suboptimal selection. Therefore, factors other than examined in our study should be taken into consideration when discussing successful salvage treatment in oral cancer. By contrast, the main strength is that patients were treated in a single institution according to a standardised protocol, thus increasing the reliability of these results. In addition, the sample size was relatively large with a long follow up.

## 5. Conclusions

The decision to perform salvage surgery in patients with recurrent oral cancer is highly challenging due to the difficulty of determining, a priori, the likelihood of surgical success, as evidenced by the 58% failure rate in our patient cohort. This is especially relevant given the negative impact of unsuccessful salvage surgery on QoL outcomes, together with the poor survival outcomes in these patients, which is comparable to survival in patients who receive palliative care alone. Given these data, it would be better to avoid salvage surgery in patients with a low probability of success. By contrast, salvage surgery should be performed when the possibility of success is high as survival rates in patients who achieve successful salvage are similar to those observed in non-recurrent cases. Additional studies are needed to identify other factors associated with successful salvage surgery in order to better stratify patients according to the likelihood of success, thus potentially avoiding the negative impact of unsuccessful salvage surgery on quality of life.

## Figures and Tables

**Table 1:** Sociodemographic and clinical characteristics of the study population.

Variable	Patients (*n* = 261) *n*, (%)	*p* Value (Recurrence Risk)
Sex		NS
Male	176 (67)	
Female	85 (33)	
Mean age (range)	59 (26–97)	NS
Primary tumour location		NS
Tongue	115 (44)	
Floor of mouth	92 (35)	
Buccal mucosa	26 (9)	
Other	27 (10)	
T classification		NS
T1	80 (30)	
T2	126 (48)	
T3	39 (16)	
T4	16 (6)	
N classification		
N0	153 (58)	<0.001
N1	50 (19)	
N2	58 (23)	
N3	0	
Stage of the disease		NS
I	63 (24.1)	
II	62 (23.7)	
III	53 (20.3)	
IV	83 (31.8)	
LVI+	21 (8)	0.046
PNI+	33 (12)	0.016
ENE+	44 (17)	0.003
Positive surgical margins	72 (27)	<0.001
Adjuvant RT	217 (83)	NS
Adjuvant CRT	71 (27)	NS

Abbreviations: NS, not significant; RT, radiotherapy; CRT, chemoradiotherapy; perineurial invasion (PNI); lymphovascular invasion (LVI); and extranodal extension (ENE).

**Table 2:** Clinical characteristics of patients with recurrent disease (*n* = 108).

Variable	Patients (108), *n* (%)
Local recurrence	40 (38)
Regional recurrence	25 (24)
Locoregional recurrence	13 (13)
Distant metastases	16 (15)
locoregional and distant	5 (4)
Second primary	7 (6)
Primary T stage	
T1	26 (24)
T2	55 (50)
T3	20 (19)
T4	7 (7)
Primary N stage	
N0	44 (40)
N1	26 (24)
N2	38 (35)
N3	0
Recurrent T stage	85 (100)
rT0	26 (30)
rT1	10 (13)
rT2	13 (15)
rT3	5 (6)
rT4	31 (36)
Recurrent N stage	85 (100)
rN0	40 (47)
rN1	13 (15)
rN2	16 (19)
rN3	16 (19)
Palliative care	72 (67)
Salvage surgery	36 (33)
Free surgical margins	22 (61)
Involved margins	14 (39)
Subsequent recurrence	21/36 (58)
Recurrent tumor location	36 (100)
Tongue	14 (39)
Floor of mouth	13 (36)
Buccal mucosa	6 (17)
Other	3 (8)

**Table 3:** Comparison of clinical factors in patients who underwent salvage surgery versus patients who received palliative care.

Primary Tumour Variable	Salvage Surgery (*n* = 36) *n*, (%)	Palliative Treatment (*n* = 72) *n*, (%)	*p* Value
Early local stage (T1–T2)	28 (77%)	53 (73%)	0.048
N0 neck	20 (55%)	24 (33%)	0.031
Free surgical margins	27 (75%)	36 (50%)	0.011
Adjuvant treatment	27 (75%)	69 (95%)	0.001
PNI+	4 (11%)	16 (22%)	NS
LVI+	2 (5%)	11 (15%)	NS
ENE+	6 (17%)	21 (29%)	NS
Mean time from primary treatment to recurrence, months	24.3	19.1	NS

Abbreviations: NS, not significant; perineurial invasion (PNI); lymphovascular invasion (LVI); and extranodal extension (ENE).

## Data Availability

The data presented in this study are available on request from the corresponding author.
